# GSK3β Plays a Negative Role During White Spot Syndrome Virus (WSSV) Infection by Regulating NF-κB Activity in Shrimp *Litopenaeus vannamei*

**DOI:** 10.3389/fimmu.2020.607543

**Published:** 2020-11-26

**Authors:** Shuang Zhang, Lulu Zhu, Cuihong Hou, Hang Yuan, Sheng Yang, Mustafa Abdo Saif Dehwah, Lili Shi

**Affiliations:** ^1^College of Fisheries, Guangdong Ocean University, Zhanjiang, China; ^2^Key Laboratory of Aquatic, Livestock and Poultry Feed Science and Technology in South China, Ministry of Agriculture, Zhanjiang, China; ^3^Aquatic Animals Precision Nutrition and High Efficiency Feed Engineering Research Center of Guangdong Province, Zhanjiang, China; ^4^Department of Medical Laboratories, Faculty of Medical and Health Science, Taiz University/AL-Turba Branch, Taiz, Yemen; ^5^Guangdong Provincial Key Laboratory of Marine Resources and Coastal Engineering, Guangzhou, China

**Keywords:** glycogen synthase kinase 3β, nuclear factor κB, negative regulation, white spot syndrome virus, *Litopenaeus vannamei*

## Abstract

Glycogen synthase kinase-3 (GSK3), a cytoplasmic serine/threonine-protein kinase involved in a large number of key cellular processes, is a little-known signaling molecule in virus study. In this study, a GSK3 protein which was highly similar to GSK3β homologs from other species in *Litopenaeus vannamei* (designated as LvGSK3β) was obtained. LvGSK3β was expressed constitutively in the healthy *L. vannamei*, at the highest level in the intestine and the lowest level in the eyestalk. White spot syndrome virus (WSSV) reduced LvGSK3β expression was in immune tissues including the hemocyte, intestine, gill and hepatopancreas. The inhibition of LvGSK3β resulted in significantly higher survival rates of *L. vannamei* during WSSV infection than the control group, and significantly lower WSSV viral loads in LvGSK3β-inhibited *L. vannamei* were observed. Knockdown of LvGSK3β by RNAi resulted in increases in the expression of LvDorsal and several NF-κB driven antimicrobial peptide (AMP) genes (including ALF, PEN and crustin), but a decrease in LvCactus expression. Accordingly, overexpression of LvGSK3β could reduce the promoter activity of LvDorsal and several AMPs, while the promoter activity of LvCactus was increased. Electrophoretic mobility shift assays (EMSA) showed that LvDorsal could bind to the promoter of LvGSK3β. The interaction between LvGSK3β and LvDorsal or LvCactus was confirmed using co-immunoprecipitation (Co-IP) assays. In addition, the expression of LvGSK3β was dramatically reduced by knockdown of LvDorsal. In summary, the results presented in this study indicated that LvGSK3β had a negative effect on *L. vannamei* by mediating a feedback regulation of the NF-κB pathway when it is infected by WSSV.

## Introduction

Shrimp is one of the main internationally traded aquatic products, which has important economic value. As an important aquaculture shrimp species, the produce of *Litopenaeus vannamei* accounts for nearly two-thirds of the total output of shrimp worldwild (1). In recent years, emerging diseases caused by various pathogens have been the main menace for sustainable development of the shrimp industry worldwide. Among these pathogens, white spot syndrome virus (WSSV) has always been a viral agent of greatest concern, although other viruses such as Taura syndrome virus (TSV), infectious hypodermal and hematopoietic necrosis virus (IHHNV) and decapod iridescent virus 1 (DIV1) were reported to cause losses in farmed shrimp (2–5). As an invertebrate, the innate immunity is the first defense line against viral infections in shrimp (6). Although a considerable number of scientific studies have focused on this for several years, knowledge of the host-virus interaction is quite insufficient. Thus, it is in urgent need to investigate the mechanisms of immune defence for disease controling in shrimp culture.

Glycogen synthase kinase 3 (GSK3), a multifunctional serine/threonine-protein kinase, initially identified as a key regulator of glycogen metabolism and insulin signaling, has been verified as being involved in various physiological and pathological processes, such as protein synthesis, signal transduction, cell proliferation and differentiation, immune response, inflammation and tumorigenesis, and so on (7, 8). GSK3 is highly conserved in species from the amoeba, *Dictyostelium discoideum*, to humans. In vertebrates, there are two isoforms of GSK3 (GSK3α and GSK3β) known, which share a highly conserved catalytic domain but differ at both termini (9). GSK3α and GSK3β perform different functions in many biological processes. GSK3α is mainly involved in glycogen metabolism, while GSK3β functions as a convergence point for multiple signaling pathways, including nuclear factor κB (NF-κB), Wnt/β-catenin, phosphatidylinositide 3-kinase/protein kinase B (PI3K/Akt), Hedgehog and STAT signaling pathways (10–12). Recent studies have unearthed GSK3β’s role in viral infections through its alteration of viral entry, replication and egress. GSK3β is known as an AKT substrate which plays a crucial role in influenza viral entry (13, 14). GSK3β silence could decrease the hepatitis C virus (HCV) replication and the production of infectious particle while GSK3β overexpression enhanced HCV replication (15). Studies *in vitro* displayed that specific inhibitors (small molecules) of GSK3β significantly decreased Tat-dependent replication of human immunodeficiency virus 1 (HIV1) (16). In addition, GSK3β inhibitors could effectively prevent the assembly and release of HCV because of the key role in HCV virion assembly and release GSK3β played, through inhibiting apolipoprotein synthesis (17). But overall, studying on the function of GSK3β and its related signaling pathways in the research field of virology is still in its initial stage, especially in invertebrates.

GSK3β has been demonstrated to play a role in the interaction between host and virus in shrimp, but studies on its specific regulation mechanism during the anti-viral immune reaction in shrimp is required, as GSK3β is a cross point in several signal pathways (18). An important pro-inflammatory pathway promoted by GSK3β is the activation of NF-κB (19). GSK3β plays a vital role in gene transcription by the way of regulating the promoter-specific recruitment of NF-κB (20). Through restraining the transcriptional activation of NF-κB, GSK3β inhibitors could lessen the inflammatory cytokine production of the TLR pathway (21). Consistently, activation of the PI3K/Akt/GSK3β signaling pathway reduced the NF-κB nuclear translocation and the pathway acts as the influencing factor in inflammatory protection (22). NF-κB plays an essential role in the antiviral immunity and is an obstacle to the survival of virus (23). The Toll and immune deficiency (IMD) pathways are Two NF-κB related signaling pathways which are considered as the main regulatory factors of the anti-viral immune response in shrimp (24). Building on the current research, the mechanism behind GSK3β mediation of NF-κB during WSSV infection in *L. vannamei* was studied in this paper. The results showed that *L. vannamei* GSK3β (LvGSK3β) has a negative effect for the shrimp during WSSV infection by regulating NF-κB activity, which is beneficial for the study on the host-virus interactional mechanisms and may offer potential strategies for disease prevention in shrimp industry.

## Materials and Methods

### Shrimp and WSSV

*L. vannamei* (approximately 4–6 g each) were purchased from a local shrimp farm in Zhanjiang City, Guangdong Province, China. As our previous study (25) described, *L. vannamei* were cultured in a recirculating water tank system for over 7 days before the experiments with water salinity and temperature maintained at 27‰ salinity at 25–27 °C. The shrimp were fed with a commercial shrimp pellet diet (Haid Group, Guangzhou, China) twice daily. At the beginning of all experimental treatments, shrimp (5% of the total) were analyzed and ensured to be free of WSSV via PCR method performed according to published standard operating procedures. The WSSV (Chinese strain, AF332093) was extracted from WSSV-infected shrimp muscle tissue and stored at −80 °C. Before injection, the muscle tissue was homogenised and prepared as a WSSV inoculum with approximately 1×10^5^ copies/µl, as previously described (26).

### Cloning the cDNA and Genome of LvGSK3β

Total RNA and the genomic DNA were extracted from the shrimp tissues using the RNeasy Mini kit (Qiagen, Hilden, Germany) and TIANGEN Marine Animals DNA Kit (TIANGEN, Guangzhou, China), respectively. First-strand cDNA synthesis was performed using a cDNA Synthesis Kit (Takara, Dalian, China) following the manufacturer’s instructions. The partial cDNA sequence of LvGSK3β was obtained from transcriptomic sequencing of *L. vannamei* (27) and its full-length cDNA sequence was cloned by a rapid amplification of cDNA ends (RACE) PCR using the SMARTer™ RACE cDNA Amplification kit (Clontech, Japan), following the user manual and the specific primers used are listed in **Table 1**. The genomic DNA sequences of LvGSK3β were obtained via PCR, according to a previously published method (28) and were based on the genome sequences of *Penaeus vannamei* breed Kehai No.1 (GenBank No. NW_020869560). The PCR products were cloned into the pMD-20 vector (Takara, Japan) and sequenced. The gene sequences obtained in this study have been deposited in the NCBI GenBank (http://www.ncbi.nlm.nih.gov/genbank/).

**Table 1:** PCR primers used in this study.

Primers	Primer sequences (5’-3’)
**cDNA cloning**	
LvGSK3β-5RACE1	TTTGCTGTGATGCCTTGCTACT
LvGSK3β-5RACE2	GCAGCACCCTCAGAAGAGTCAA
LvGSK3β-3RACE1	TATAAACCTGGTGTCACGACTG
LvGSK3β-3RACE2	GACTACCCAACAATCGGGAGTT
**Genomic DNA cloning**	
GLvGSK3β-1F	AGTCTTGAGCCAGGTCTGTCGT
GLvGSK3β-1R	ATTTGTTCTCTTTACCCATACACTCA
GLvGSK3β-2F	TCTCATACATCGTCCTCGGTCTT
GLvGSK3β-2R	TGTGTGGTGGACTCGGCTG
GLvGSK3β-3F	AAGATTACCACAGTTATTGCCACC
GLvGSK3β-3R	CCTTTCGTTGTCACATCTTCATC
GLvGSK3β-4F	TAAAGTAGCAAGGCATCACAGCA
GLvGSK3β-4R	AATAGCGGGAGCAGATGTACG
GLvGSK3β-5F	GTTGTTCCGAAGCCTTGCCT
GLvGSK3β-5R	GCAGGCACAGTCTTGTCAGC
GLvGSK3β-6F	TCTTTCAGTGCTGCCCTTGT
GLvGSK3β-6R	CCCTCAGAAGAGTCAACGGC
**Genome walking**	
AP1	GTAATACGACTCACTATAGGGC
AP2	ACTATAGGGCACGCGTGGT
LvGSK3β-GWR1	CAGCCTTTCCGAGATGCG
LvGSK3β-GWR2	GTCGCTGCGGAGTCAAAGAA
**qPCR**	
qLvEF1α-F	GAAGTAGCCGCCCTGGTTG
qLvEF1α-R	CGGTTAGCCTTGGGGTTGAG
qLvGSK3β-F	GGTGGGAGTGGGGAGGGT
qLvGSK3β-R	TCCTTCCAGCCTCATTGTTGTG
qLvDorsal-F	AGATGGAATGATAGAATGGGAAGC
qLvDorsal-R	GTACACCTTTATGGGGTTCTCTATCTC
qLvCactus-F	GGAGGCGTGCCAGTGACTATG
qLvCactus-R	GAAGTAACGATCTGCATTGAAGGG
qLvALF1-F	GGATGTGGTGTCCTGGATGG
qLvALF1-R	GCGTCGTCCTCCGTGATG
qLvALF2-F	GCGAACAAACTCACTGGACTG
qLvALF2-R	ACATGCGACCCTGGAATACAG
qLvALF3-F	GACCTGTCCAACCCTGAGC
qLvALF3-R	TCGCCTCCTCCTCCGTTATC
qLvPEN2-F	TTCTCAGATGTCCGCATTTGC
qLvPEN2-R	ACGTTGTCAAGCCAGGTTTCC
qLvPEN3-F	TACAACGGTTGCCCTGTCTCA
qLvPEN3-R	ACCGGAATATCCCTTTCCCAC
qLvPEN4-F	GGTGCGATGTATGCTACGGAA
qLvPEN4-R	CATCGTCTTCTCCATCAGCCA
qLvCru1-F	GTAGGTGTTGGTGGTGGTTTC
qLvCru1-R	CTCGCAGCAGTAGGCTTGAC
qLvCru2-F	GGTACGTCTGCTGCAAGCC
qLvCru2-R	CTGAGAACCTGCCACGATGG
qLvCru3-F	TCCACAATGGTCAGCGTCAAG
qLvCru3-R	CTGTCCGACAAGCAGTTCCTC
WSSV32678-F	TGTTTTCTGTATGTAATGCGTGTAGGT
WSSV32753-R	CCCACTCCATGGCCTTCA
TaqMan probe-WSSV32706	CAAGTACCCAGGCCCAGTGTCATACGTT
**dsRNA synthesis**	
dsLvGSK3β-T7F^a^	TAATACGACTCACTATAGGAGGTGCTTCAGGACAAACGC
dsLvGSK3β-T7R^a^	TAATACGACTCACTATAGGTAGCGGGAGCAGATGTACGATA
dsLvGSK3β-F	AGGTGCTTCAGGACAAACGC
dsLvGSK3β-R	TAGCGGGAGCAGATGTACGATA
dsLvDorsal-T7F^a^	TAATACGACTCACTATAGGGTAGGATACAAAGGACCTGCTG
dsLvDorsal-T7R^a^	TAATACGACTCACTATAGGTGGAAATCACCGAAGGCT
dsLvDorsal-F	GTAGGATACAAAGGACCTGCTG
dsLvDorsal-R	TGGAAATCACCGAAGGCT
dsGFP-T7F^a^	TAATACGACTCACTATAGGATGGTGAGCAAGGGCGAGGAG
dsGFP-T7R^a^	TAATACGACTCACTATAGGTTACTTGTACAGCTCGTCCATGCC
dsGFP-F	ATGGTGAGCAAGGGCGAGGAG
dsGFP-R	TTACTTGTACAGCTCGTCCATGCC
**EMSA probes**	
Bio-probe1	CCTTTCCAATGAAACAGAAAAACCTCGCGCACAGACGA
Unbio-probe1	CCTTTCCAATGAAAGCGCACAGACGACTCCCGAC
Bio-probe2	TAGCGATGAATGGGGTGTGTTTCACTGTGAGAATCTT
Unbio-probe2	TAGCGATGAATGGTGTGAGAATCTTGATTTTGGATGCT
**Protein expression**	
pAcLvGSK3β-F^b^	CCG**CTCGAG**ATGAGTGGACGACCCAGGACT
pAcLvGSK3β-R^b^	CGG**GGGCCC**ATTATCATTTACAGCAGCAGCT
pAcLvDorsal-F^b^	GGGGTACC**ATCAAA**ATGTTTGTTGCCCAGCGTACTTC
pAcLvDorsal-R^b^	TT**GGGCCC**ATATCAGAAAATATCCAAAACTTACCC
pAcLvCactus-F^b^	GGGGTACC**ATCAAA**ATGTGGCACATTGGCAGTGCCCA
pAcLvCactus-R^b^	TT**GGGCCC**GAAGTAACGATCTGCATTGAAGG

a T7 RNA polymerase promoter sequence are underlined.

b Nucleotides in bold represent the restriction sites introduced for cloning.

### Genomic Walking

The 5’ flanking regulatory regions of LvGSK3β were cloned by genome walking PCR amplification via the GenomeWalker Universal Kit (Clontech, Dalian, China), according to the methods of a previous paper (29). Two pairs of primers, AP1/LvGSK3β-R1 and AP2/LvGSK3β-R2, were used to perform the primary and nested genome walking PCR amplification. The PCR products were cloned into the pMD-20T vector (Takara, Dalian, China) and sequenced. Primers are listed in **Table 1**.

### Bioinformatics Analyses

The protein domain topologies and the genome sequences were analysed using the SMART program (http://smart.embl-heidelberg.de/) and Splign program (https://www.ncbi.nlm.nih.gov/sutils/splign/splign.cgi). Protein sequences of GSK3β homologues from other species were selected from the NCBI databases. Multiple sequence alignments were performed using the ClustalX 2.0 program (http://www.ebi.ac.uk/tools/clustalw2) and visualized using GeneDoc. The similarities among the GSK3β proteins were calculated in GeneDoc. The neighbor-joining phylogenetic trees were constructed based on the amino acid sequences using MEGA 5.0 (http://www.megasoft-ware.net/download_form), applying the Poisson distribution substitution and bootstrapping procedure with a minimum of 1,000 bootstraps. The LvGSK3β promoter sequence was analyzed using Consite (http://consite.genereg.net/).

### Tissue Distribution, Immune Challenge, and Gene Expression Analyses

Eleven tissues, including the hemocytes, hepatopancreas, gill, heart, stomach, pyloric caecum, nerve, epithelium, eyestalk, intestine and muscle, were obtained from 10 healthy *L. vannamei* for RNA extraction. For the challenge experiments, 60 healthy *L. vannamei* divided into two groups (n = 30) were intramuscularly injected with WSSV (10^6^ copies/g) in the third abdominal segment. *L. vannamei* injected with PBS were used as controls. At 0, 4, 8, 12, 24, 48, and 72 h post-injection (hpi), three animals from each group were randomly sampled for hemocyte, hepatopancreas, gill and intestine collection. Total RNA extraction, reverse transcription and real-time PCR (qPCR) analysis were performed as detailed in previous research (30). The qPCR was performed in a Roche Light Cycler480 thermal cycler (Roche Applied Science, Germany). Elongation factor 1α (EF1α, GenBank accession No. GU136229) was used as the internal control. The expression level of LvGSK3β was calculated using the Livak(2^−ΔΔCt^) method after normalization to LvEF1α. All samples were tested in triplicate. Primer sequences are listed in **Table 1**.

### RNA Interference

As described in our previous studies (31, 32), the double-stranded RNAs (dsRNAs) specific to LvGSK3β, LvDorsal and GFP (as control) were synthesized using an *in vitro* transcription method, by using the T7 RiboMAX™ Express RNAi System (Promega, USA), and the quality and amount of dsRNA were checked after annealing via gel electrophoresis. For the RNA interference efficiency determination, healthy *L. vannamei* were injected with 50 µl PBS containing 5 µg LvGSK3β, LvDorsal or GFP dsRNA. At 24 and 48 hpi, the hemocytes from each group were sampled for qPCR to detect the RNAi efficiency, the effects of LvGSK3β on the expression of LvDorsal, LvCactus and nine antimicrobial peptides (AMP) genes and the effects of LvDorsal on the expression of LvGSK3β in *L. vannamei*. Primer sequences are listed in **Table 1**.

### WSSV Challenge Experiments in LvGSK3β-Inhibited *L. vannamei*

LvGSK3β was inhibited both on the enzyme activity and mRNA expression levels by injecting with the specific inhibitor and dsRNA. Specific inhibitor of LvGSK3β, named TWS-119 (Selleck Chemicals, USA) was used with the dose refer to the Inhibitory concentration 50 (IC50) (33). At 4, 8 and 12 hpi, the hemocytes from each group were sampled for the detection of inhibition efficiency of LvGSK3β activities, using the GSK3β Kinase Activity Quantitative Detection Kit (Genmed, China). The expression of LvGSK3β was inhibited by RNAi as described above in Section *RNA Interference*. For the mortality test, *L. vannamei* were injected with 50 µl PBS containing or not containing 10^6^ WSSV after 8 h of TWS-119 injection or 48 h of dsRNA injection. Cumulative mortality was recorded every 4 h and the differences between groups were analysed using the Mantel–Cox (log-rank *χ*^2^ test) method with the software GraphPad Prism. To investigate the genome copies of WSSV, total DNA was extracted from muscle at 48, 72 and 96 hpi and absolute real-time PCR was performed using primers WSSV32678-F/WSSV32753-R and a Taq Man fluorogenic probe, as in a previous study (34).

### Dual-Luciferase Reporter Assays

The gene-specific primers were designed to amplify the ORF of LvGSK3β without introducing a stop codon. The amplified product was cloned into a pAc5.1/V5-His A vector (Invitrogen) to allow subsequent expression of V5-tagged LvGSK3β proteins. All of the vectors with reporter genes used in this paper, including Dorsal, Cactus and AMPs in shrimp or *Drosophila*, were obtained from our previous studies (30, 35). Since no permanent shrimp cell line was available, *Drosophila* Schneider 2 (S2) cells were cultured for promoter activity analysis at 28 °C in Schneider’s Insect Medium (Sigma, USA) containing 10% fetal bovine serum (Gibco, USA) for the *in vitro* experiments. After 24 h of culturing in a 96-well plate, the S2 cells of each well were transfected with 0.05 μg of firefly luciferase reporter-gene plasmids, 0.01 μg of pRL-TK renilla luciferase plasmid (Promega, as an internal control) and 0.05 μg of (otherwise indicated) either pAc-LvGSK3β-V5 plasmids or pAc5.1-Basic plasmids (control). Each experiment was repeated in 6 wells. 48 hours post-transfection, the activity of the luciferases was detected in order to calculate the relative ratios of firefly and renilla luciferase activity using the Dual-Glo^®^ Luciferase Assay System kit (Promega, USA), according to the manufacturer’s instructions.

### Electrophoretic Mobility Shift Assay

An electrophoretic mobility shift assay (EMSA) was performed using a Light Shift Chemiluminescent EMSA kit (Thermo Fisher Scientific, Waltham, MA, USA) according to a previously published method (36). Briefly, the biotin-labeled or unbiotin-labeled probes were designed using the Dorsal binding motif sequence. The mutant probe was designed by deleting the Dorsal-binding motif sequence. All of the probes were synthesized by Life Technologies (Shanghai, China) and the sequences are listed in **Table 1**. RHD domain of Dorsal (Dorsal-RHD) was cloned into the modified pGEX-4T plasmid to get rDorsal-RHD-GST. GST tag of rDorsal-RHD-GST was then removed using rPorcine Enterokinase from Glutathione Resin Kit. The purification productions of rDorsal-RHD-GST and rDorsal-RHD were analyzed using SDS-PAGE and stained with Coomassie blue. Purified rDorsal-RHD protein (10 μg) was incubated with 20 fmol of the probes for the binding reactions. The reactions were separated on a 5% native PAGE gel, transferred to positively charged nylon membranes (Roche, Germany) and cross-linked by UV light. Then, the biotin-labeled DNA on the membrane was detected by chemiluminescence and developed on X-ray film, followed by enhanced chemiluminescence (ECL) visualisation (Tanon, Shanghai, China).

### Co-Immunoprecipitation Assays

Co-immunoprecipitation (Co-IP) assays were performed as described in the previous study (36). To investigate the interaction of LvGSK3β with LvDorsal or LvCactus, pAc-LvGSK3β-GFP or pAc5.1-GFP (control) were co-transfected with individual plasmids along with pAc-LvDorsal-HA or pAc-LvCactus-HA into the S2 cells. Forty eight hours post transfection, cells were harvested and washed with ice-cold PBS three times, and then lysed in IP Lysis Buffer with a protease inhibitor cocktail (Sigma, USA). Both Co-IP and reciprocal Co-IP experiments were carried out using anti-HA affinity gel (Sigma, USA) and samples were subjected to SDS-PAGE assay. The precipitated protein was examined using western-blots, with rabbit anti-GFP antibody as the primary antibody, and alkaline phosphatase-conjugated goat anti-rabbit as the secondary antibody (Sigma, USA). A standardized aliquot (5%) of each total input cell lysates was also examined as control.

### Statistical Analyses

The relative gene expression data were statistically analysed and all data were presented as mean ± SD. Student’s t-tests were used to make comparisons among groups. For the mortality analysis, the data were statistically analysed using GraphPad Prism (Graphpad, San Diego, CA, USA) to obtain Kaplan–Meier plots (log-rank *χ*^2^ test).

## Results

### Bioinformatics Analyses of LvGSK3β

**Figure 1:**
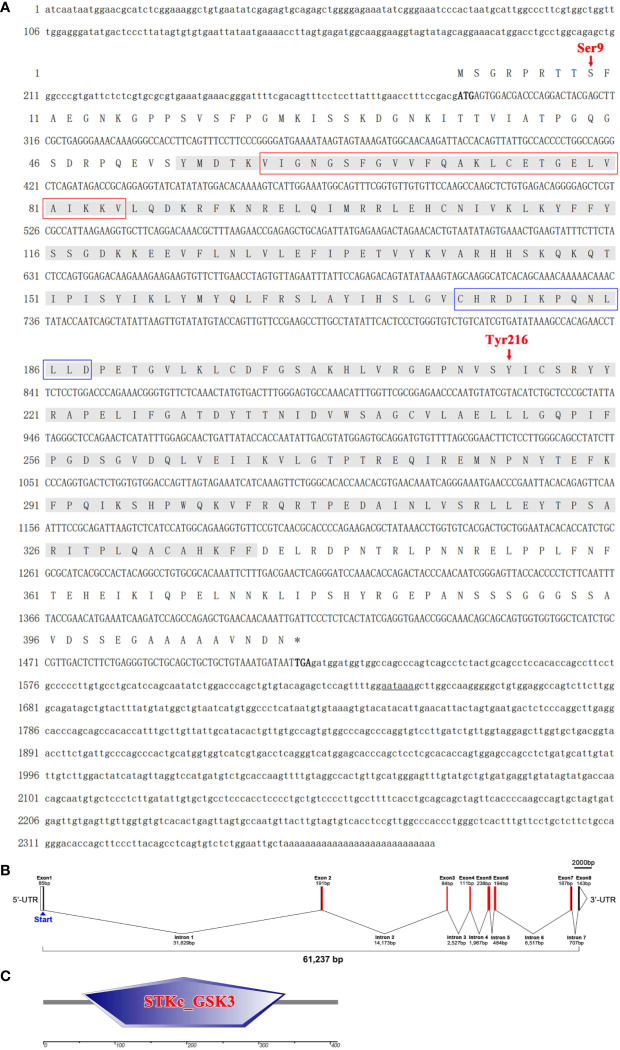
The sequence analysis of LvGSKβ. **(A)** The full-length cDNA sequence and deduced amino acid sequences of LvGSKβ. The ORF of the nucleotide sequence was shown in upper-case letters, while the 5’ and 3’-UTR sequences were shown in lowercase. The nucleotide (lower row) and deduced amino acid (upper row) sequences are shown and numbered on the left. The translation initiation codon (ATG) and stop codon (TAA) are in bold. The STKc_GSK3 domain is shaded and protein kinase ATP-binding region, serine/threonine protein kinases active site and two candidate regulatory residues (Ser9 and Tyr216) are marked out. The poly A signal (aataaa) are underlined. **(B)** Gene structure of LvGSKβ. Boxes in represent exons, and lines in represent introns. The numbers indicate the length (bp) of the exons and introns. **(C)** Structural features of LvGSKβ. Conserved domains of LvGSKβ are predicted by SMART program.

As shown in **Figure 1A**, the transcript of LvGSK3β was 2,382 bp in length, with a 286 bp 5'-untranslated region (UTR), an 863 bp 3'-UTR containing a poly (A) tail and a 1233 bp open reading frame (ORF) encoded a protein of 410 amino acids at the calculated molecular weight of 46,098.78 Da and an estimated pI of 8.70 (GenBank No. KU641425). Additionally, a protein kinase ATP-binding region signature (between amino acids 61 and 85), a serine/threonine protein kinases active site signature (between amino acids 176 and 188) and two candidate regulatory residues at positions 9 (serine) and 216 (tyrosine) were identified. The genomic sequence of LvGSK3β (GenBank No. MT891030) was also obtained, showing that the LvGSK3β gene consists of eight exons and seven introns (**Figure 1B**). Analysis of the genome sequences showed that all of the exon-intron boundaries in LvMyD88 conform to the consensus GT/AG rule for splicing (37). SMART analysis revealed that the LvGSK3β protein contained one protein kinase domain named STKc_GSK3 between residues 54 and 338 (**Figure 1C**).

**Figure 2:**
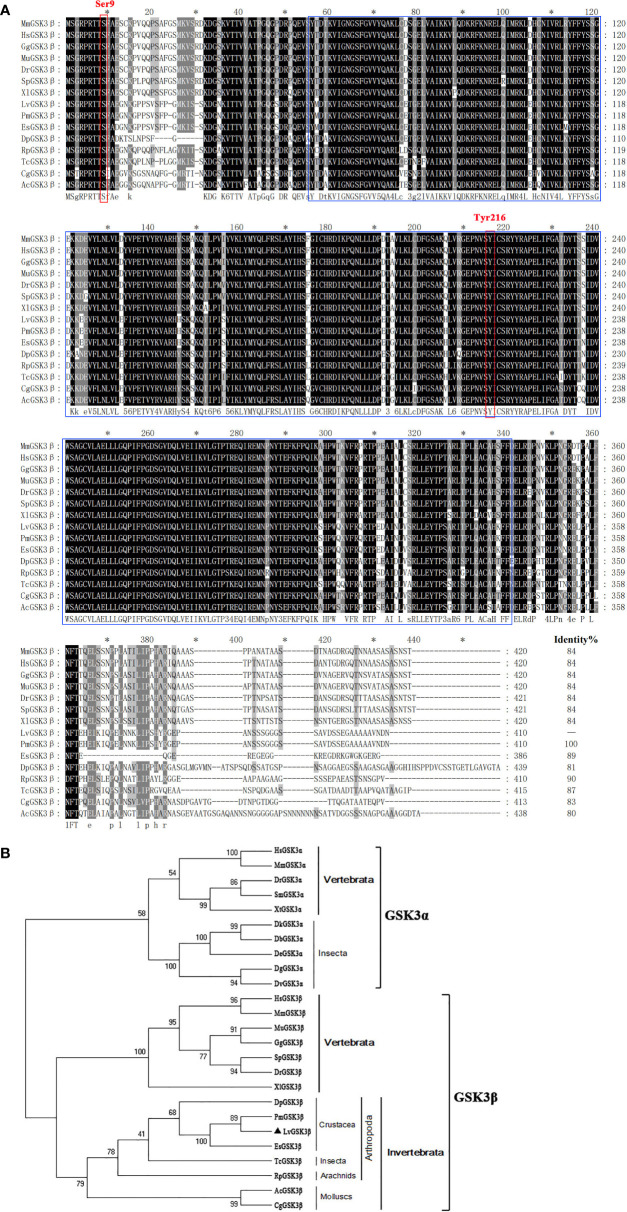
Multiple sequence alignment and phylogenetic tree analysis of GSK3β from *L. vannamei* and other species. **(A)** Multiple sequence alignment of GSK3β proteins. The amino acid sequences of GSK3β from typical organisms were aligned using the ClustalX 2.0 program. The black shade represent 100% identity, dark gray represented 80% identity, light gray represented 60% identity. The region of STKc_GSK3 domain was boxed in blue and two candidate regulatory residues Ser9 and Tyr216 were boxed in red. **(B)** Neighborjoining phylogenetic tree analysis. A rooted tree was constructed via the neighbor-joining method and was bootstrapped 1000 times using MEGA 5.0 software (http://www.megasoftware.net/index.html). LvGSK3β is denoted by ▲. The genes used are listed as follows, GSK3α isoform: HsGSK3α, *Homo sapiens* GSK3α (Accession No. NP_001139628); MmGSK3α, *Mus muscμlus* GSK3α (Accession No. AAD39258); DrGSK3α, *Danio rerio* GSK3α (Accession No. NP_571465); DrGSK3α, *Scophthalmus maximus* (Accession No. AWP21281); DrGSK3α, *Xenopus tropicalis* (Accession No. XP_002936450); DbGSK3α, *Drosophila bipectinata* (Accession No. XP_017108632); DeGSK3α, *Drosophila elegans* (Accession No. XP_017115500); DtGSK3α, *Drosophila takahashii* (Accession No. 017013230); DvGSK3α, *Drosophila virilis* (Accession No. XP_002051677); DgGSK3α, *Drosophila grimshawi* (Accession No. XP_001988855); GSK3β isoform: LvGSK3β, *L. vannamei* GSK3β (Accession No. KU641425); HsGSK3β, *Homo sapiens* GSK3β (Accession No. AAH00251); MmGSK3β, *Mus muscμlus* GSK3β (Accession No. AAD39258); MuGSK3β, *Melopsittacus undulatus* GSK3β (Accession No. XP_005143085); GgGSK3β, *Gallus gallus* GSK3β (Accession No. XP_004938236); SpGSK3β, *Schizothorax prenanti* GSK3β (Accession No. AKA09693); DrGSK3β, *Danio rerio* GSK3β (Accession No. NP_571456); XlGSK3β, *Xenopus laevis* GSK3β (Accession No. AAA84444); DpGSK3β, *Daphnia pulex* GSK3β (Accession No. EFX72595); PmGSK3β, *Penaeus monodon* GSK3β (Accession No. QIK00339); EsGSK3β, *Eriocheir sinensis* GSK3β (Accession No. ANZ22981); TcGSK3β, *Tribolium castaneum* GSK3β (Accession No. XP_008192199); RpGSK3β, *Rhipicephalus pulchellus* GSK3β (Accession No. JAA60305); AcGSK3β, *Aplysia californica* GSK3β (Accession No. XP_005107895); CgGSK3β, *Crassostrea gigas* GSK3β (Accession No. EKC35379).

**Figure 3:**
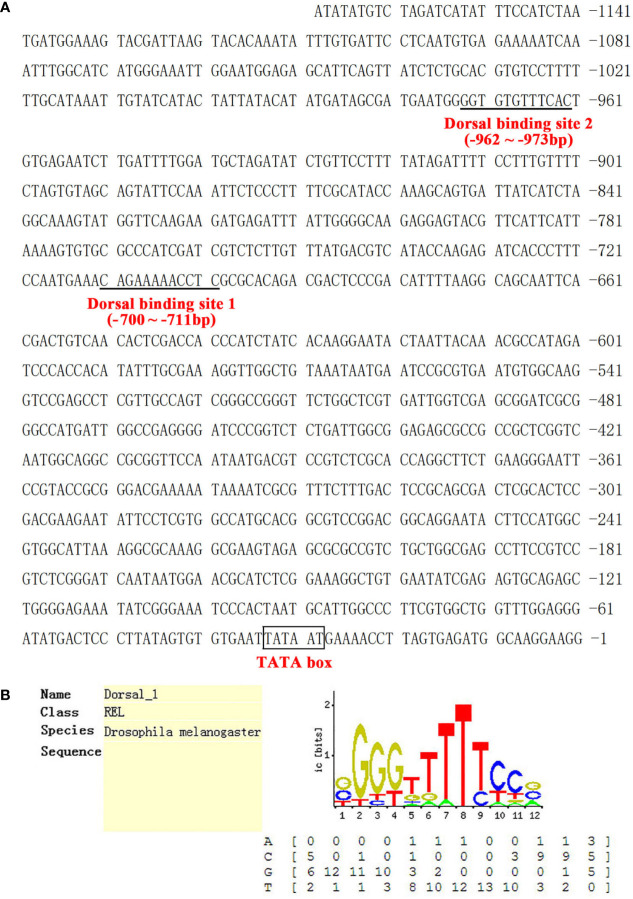
The sequence analysis of the LvGSK3β promoter. **(A)** The promoter sequence of LvGSK3β. The predicted TATA-box was boxed and the putative Dorsal binding sites were underlined. **(B)** Prediction of the Dorsal binding sites sequence in LvGSK3β promoter using Consite software.

Multiple sequence alignment indicated that LvGSK3β shares a high similarity of more than 80% with GSK3β proteins from invertebrates to vertebrates, with the lowest homology (80%) to GSK3β from *Aplysia californica* and the highest homology (100%) to GSK3β from *Penaeus monodon* (**Figure 2A**), which is also a shrimp species. The neighbour-joining (NJ) phylogenetic tree showed that GSK3α and GSK3β were closely related to their own homologues from other species. All of the GSK3β proteins used in this study were separated into five clades including the crustacea, insecta, arachnids, molluscs and vertebrates groups (**Figure 2B**). GSK3β from *L. vannamei*, *P. monodon*, *Daphnia pulex* and *Eriocheir sinensis* were clustered together in the crustacea group. *L. vannamei* GSK3β and *P. monodon* GSK3β showed the closest evolutionary relationship. As shown in **Figure 3A**, the promoter region of LvGSK3β (1,169 bp in length) contained two potential Dorsal binding sites (−700 to −711 bp and −962 to −973 bp) (**Figure 3B**).

### Expression Profiles of LvGSK3β in Healthy and WSSV Challenged *L. vannamei*

**Figure 4:**
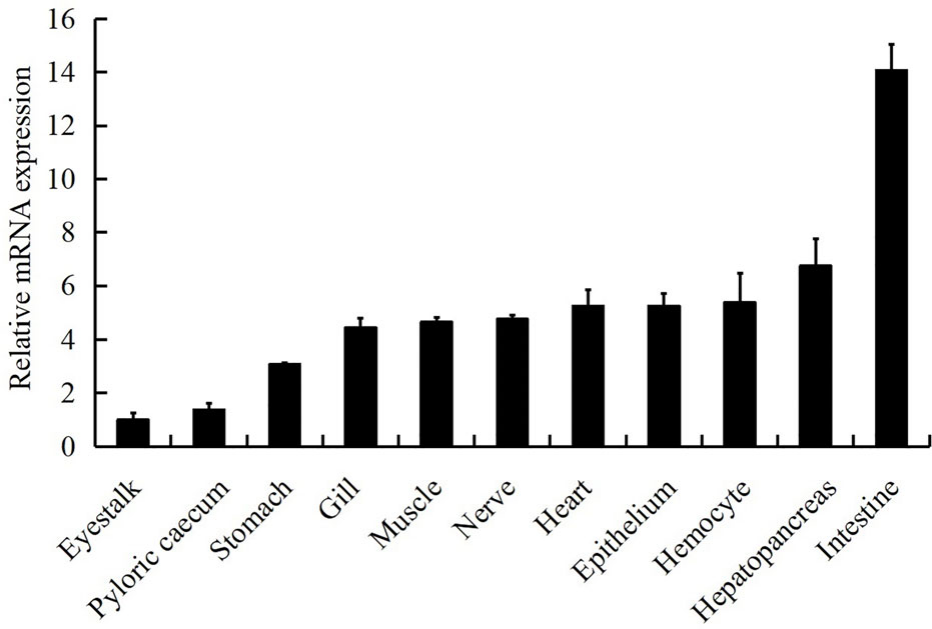
Tissue distribution of LvGSK3β expression in healthy *L. vannamei*. Ten animals were used for tissue sampling. The expression of LvGSK3β in eyestalk was set as 1.0. LvEF1α was used as the internal control to normalize the cDNA template used for qPCR analysis. The results were based on three independent experiments and expressed as mean values ± SD.

LvGSK3β was detected in all of the examined tissues (**Figure 4**). The relative expression levels of LvGSK3β in the other tissues were normalised to that in eyestalk, which was the lowest and set as the baseline (1.0). LvGSK3β was expressed highest in the intestine, in which the expression level of LvGSK3β was 14.07-fold over the baseline. As shown in **Figure 4**, LvGSK3β expression in the gill, muscle, nerve, heart, hepatopancreas and hemocyte were all nearly 5.00-fold over the baseline and not significantly different from each other.

**Figure 5:**
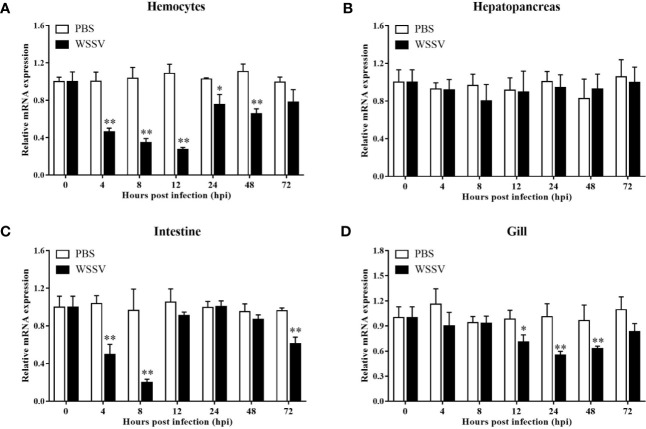
Expression profiles of LvGSK3β in haemocytes **(A)**, hepatopancreas **(B)**, gill **(C)** and intestine **(D)** from WSSV or PBS challenged *L. vannamei*. qPCR was performed in triplicate for each sample. Expression values were normalized to those of LvEF1α using the Livak (2^−ΔΔCt^) method and the data were provided as the means ± SD of triplicate assays. The statistical significance was calculated using Student’s t-test (*p < 0.05, **p < 0.01).

Using the four important immune tissues, including hemocytes, hepatopancreas, intestine and gill, LvGSK3β expression after WSSV challenge was investigated using qPCR (**Figure 5**). The expression of LvGSK3β was reduced to different degrees by WSSV challenge in the haemocytes, intestine and gill but not in the hepatopancreas. In the hepatopancreas there was no significant difference in LvGSK3β expression between the infection group and the control group at all of the sampled timepoints (**Figure 5B**). In the hemocyte, LvGSK3β expression was significantly down-regulated starting at 4 hpi, reaching a low-point at 12 hpi at 25.21% of the control. At 72 hpi, LvGSK3β expression recovered to a level not perceptibly different from the control (**Figure 5A**). In the intestine, the expression of LvGSK3β in the infection group was notably lower than the control group at 4, 8 and 72 hpi, with the lowest level at 8 hpi at 21.00% of the control (**Figure 5C**). When compared with the control group, LvGSK3β expression in the gill of the infection group was significantly decreased at 12, 24 and 48 hpi by 72.02%, 54.78% and 65.16%, respectively (**Figure 5D**).

### Function of LvGSK3β in WSSV Infection

**Figure 6:**
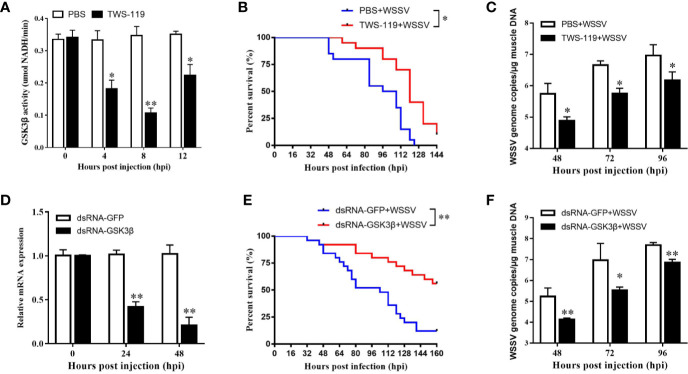
Functional analysis of LvGSK3β in WSSV infection. The inhibiting efficiencies of LvGSK3β by TWS-119 **(A)** or dsRNA-LvGSK3β **(C)** injection. The detection of LvGSK3β activities and expression was performed in triplicate for each sample. Expression values were normalized to those of LvEF1α using the Livak (2^−ΔΔCt^) method and the data were provided as the means ± SD of triplicate assays. Cumulative mortalities of TWS-119 **(B)** and dsRNA-LvGSK3β **(D)** treated *L. vannamei* during WSSV infection. The experiments were performed two times with identical results. Differences in cumulative mortality levels between treatments were analyzed by using the log-rank *χ*^2^ test (*p <0.05, **p <0.01). WSSV genome copies in muscle tissue of TWS-119 **(C)** and dsRNA-LvGSK3β **(E)** treated *L. vannamei* post WSSV infection. Absolute real-time PCR was performed using primers WSSV32678-F/WSSV32753-R and a Taq Man fluorogenic probe. Each bar represented the mean ± SD of three samples. Statistically significant differences were represented with asterisks (**p < 0.01 and * *p* < 0.05).

Refer to IC50 of TWS-119 in animals (33), a dose of 5 µg/g TWS-119 per shrimp was chosen for the LvGSK3β-inhibition test. As **Figure 6A** shows, the enzyme activity of LvGSK3β was significantly reduced at 4, 8 and 12 hpi, with the lowest level at 8 hpi, which was the timepoint chosen for the following WSSV challenge experiment. In the TWS-119 + WSSV group, the survival rates of *L. vannamei* were notably higher than that of the PBS + WSSV group starting at 56 hpi (**Figure 6B**). At 124 hpi, the survival rate of *L. vannamei* in the TWS-119 + WSSV group was 43.60% but the *L. vannamei* in the PBS+WSSV group had all died. The final survival rate of *L. vannamei* at 144 hpi was 10.30% for the TWS-119 + WSSV group. Consistently, the WSSV genome copies in the muscle of the TWS-119 injected *L. vannamei* at 48, 72 and 96 hpi were significantly less than those in the PBS injected *L. vannamei* (**Figure 6C**).

RNAi was also performed to investigate the role of LvGSK3β during WSSV infection. qPCR was used to check the silencing efficiency of LvGSK3β. As shown in **Figure 6D**, at 48 h after injection of LvGSK3β dsRNA, the expression levels of LvGSK3β at 24 and 48 hpi were significantly down-regulated to ~0.43-fold and ~0.21-fold of the GFP dsRNA injection group, respectively. Similarly as for the results when LvGSK3β were inhibited by TWS-119, the survival rates of *L. vannamei* in the LvGSK3β dsRNA + WSSV group were also significantly higher than that of the GFP dsRNA + WSSV group, and the final survival rate of *L. vannamei* at 144 hpi was 53.85 and 14.29% for the TWS-119 + WSSV group and GFP dsRNA + WSSV group, respectively (**Figure 6E**). Furthermore, the viral loads of the LvGSK3β dsRNA + WSSV group were significantly lower than those of the GFP dsRNA + WSSV control group, with a ~15.52-fold, ~40.78-fold and~7.06-fold decrease at 48, 72 and 96 h, respectively (**Figure 6F**).

### Influence of LvGSK3β on Expression of NF-κB Related Genes in *L. vannamei*

**Figure 7:**
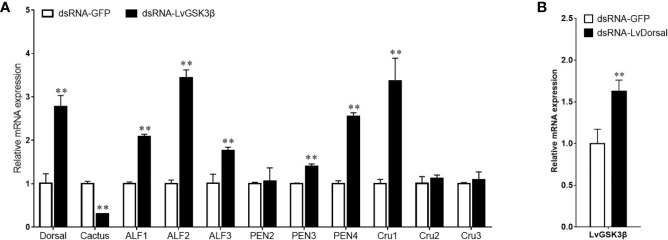
Influence of LvGSK3β on expression of NF-κB related genes **(A)** and LvDorsal on expression of LvGSK3β **(B)** in *L. vannamei* The mRNA levels of NF-κB related genes and LvGSK3β in *L. vannamei* at 48 h post dsRNA injection were detected using qPCR with LvEF1α gene as internal control. For each gene, the value in the dsRNA-GFP injected sample was set as the baseline (1.0) for comparison (**p < 0.01).

NF-κB has important roles in the regulation of AMP genes in shrimp (2). Considering the fact that the promoter of LvGSK3β contained two potential Dorsal binding sites, the expression of NF-κB related genes (Dorsal, Cactus and AMP genes) in LvGSK3β dsRNA injected shrimp were investigated to primarily explore the underlying mechanism of LvGSK3β during WSSV infection. When compared with the control GFP dsRNA, the LvGSK3β dsRNA significantly increased LvDorsal expression but decreased LvCactus expression in the haemocyte of *L. vannamei*. Consistent with this, the expression of several AMP genes, including three antilipopolysaccharide factors (LvALF1, LvALF2, LvALF3), two penaeidins (LvPEN3, LvPEN4) and one crustin (LvCRU1) were significantly up-regulated at 48 h after injection with LvGSK3β dsRNA (**Figure 7A**).

### Influence of LvDorsal on Expression of LvGSK3β in *L. vannamei*

The expression of LvGSK3β in LvDorsal dsRNA injected shrimp was also investigated. When LvDorsal was knocked down by dsRNA injection, the expression of LvGSK3β in the hemocyte of *L. vannamei* was significantly higher than the control GFP dsRNA injected group (**Figure 7B**).

### Regulatory Effects of LvGSK3β on the Promoter Activities of NF-κB Related Genes

**Figure 8:**
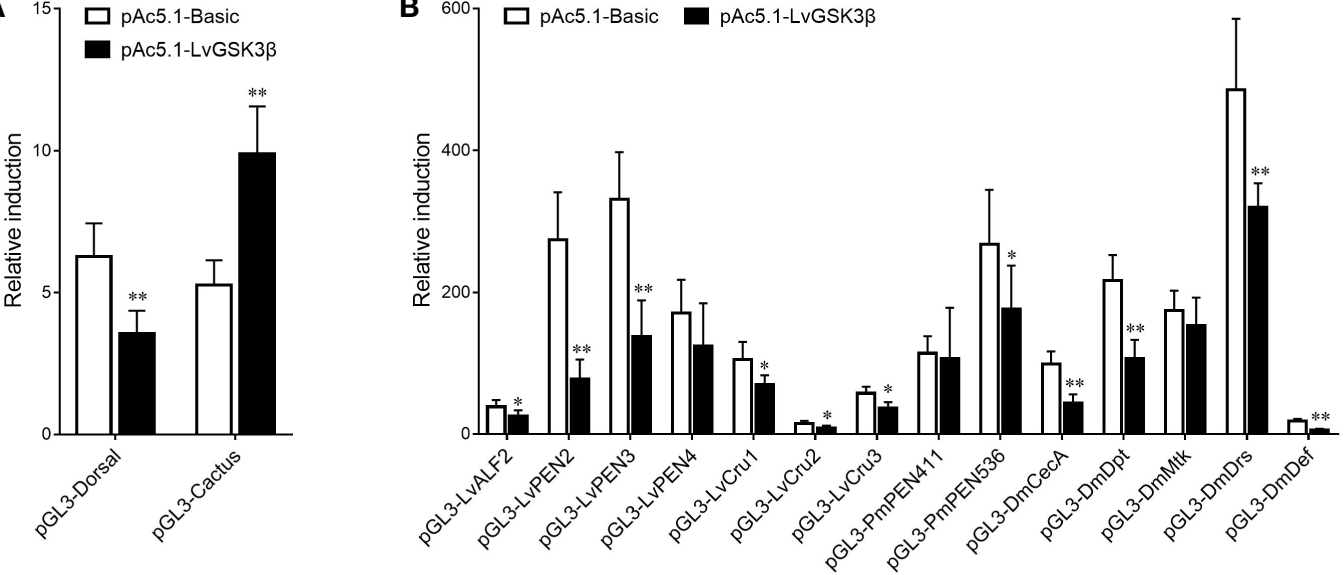
Effects of LvGSK3β on the promoter activities of NF-κB related genes in *L. vannamei*. **(A)** LvDorsal and LvCactus promoters; **(B)** AMPs promoters. *Drosophila* S2 cells were transfected with the protein expression vectors (LvGSK3β and the pAc5.1 empty vector as a control), the reporter gene plasmid, and the pRL-TK Renilla luciferase plasmid (as an internal control). After 48 h, the cells were harvested for measurement of luciferase activity using the dualluciferase reporter assay system (Promega). The bars indicated the mean ± SD of the luciferase activity (n = 6). The statistical significance was calculated using Student’s t-test (*p < 0.05, **p < 0.01).

Previous results *in vivo* showed that LvGSK3β could regulate the expression of NF-κB related genes, so the effects of LvGSK3β on the promoter activities of NF-κB related genes were further explored *in vitro* using dual-luciferase reporter assays. As shown in **Figure 8**, overexpression of LvGSK3β dramatically down-regulated the promoter activities of LvDorsal (56.61%) and the AMP genes including the *L. vannamei* AMPs LvALF2 (65.80%), LvPEN2 (28.29%), LvPEN3 (41.18%), LvCru1 (65.96%), LvCru2 (59.04%) and LvCru3 (64.23%), the *Penaeus monodon* AMP PmPEN536 (66.05%), and the *Drosophila* AMPs, DmCecA (44.37%), DmDpt (49.16%), DmDrs (65.76%) and DmDef (21.23%). However, the promoter activity of LvCactus was significantly induced by ~1.88 fold.

### Interaction Between LvDorsal and the LvGSK3β Promoter

**Figure 9:**
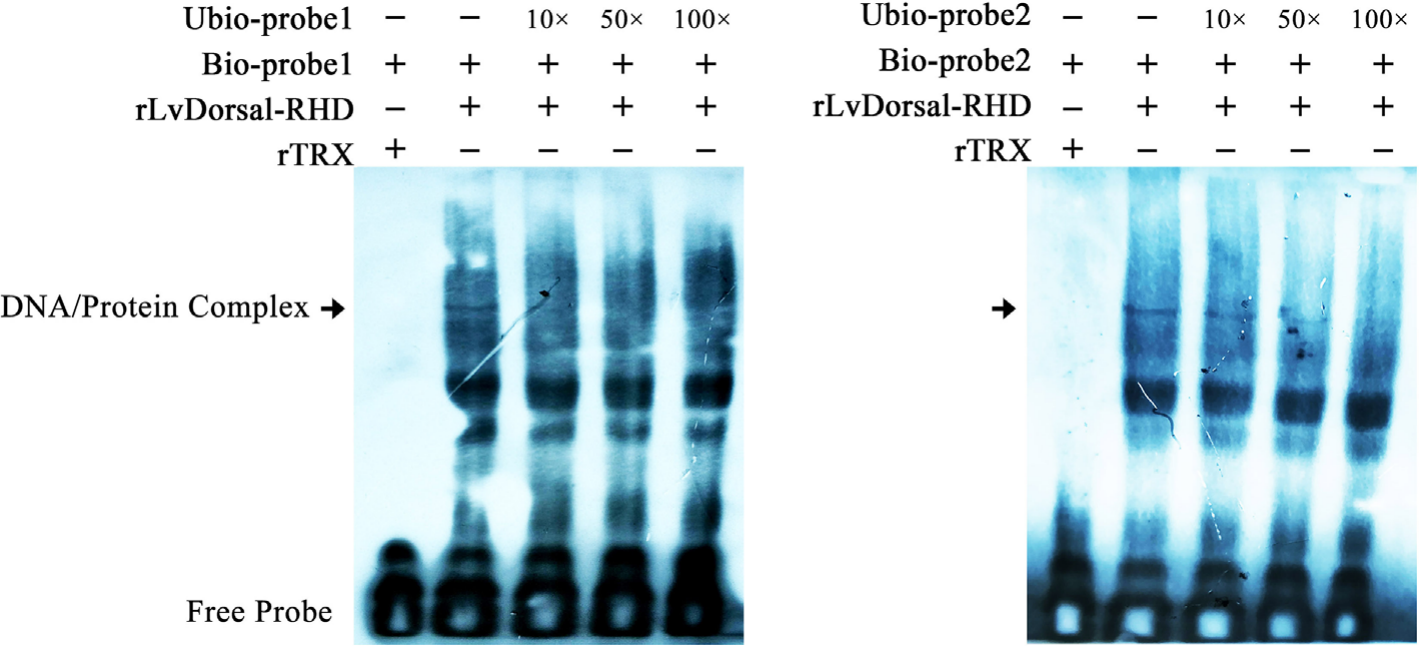
Dorsal interacted with the predicted binding motifs of LvGSK3β promoter *in vitro*. An EMSA was performed using biotin-labeled (Bio-) or unlabeled (Unbio-) probes containing or not containing the Dorsal binding motifs of LvGSK3β. Biotin-labeled or mutated biotin-labeled dsDNA probes were incubated with 10 μg of purified rLvDorsal-RHD protein. Unlabeled probe was added to compete with binding, and an rTrx protein was used as a control. Probe1 and probe 2 referred to the Dorsal binding motif 1 and 2, respectively. All experiments were performed three times with similar results.

To further investigate the interaction between LvDorsal and the LvGSK3β promoter, EMSA was performed using the purified 6His-tagged RHD domain of Dorsal protein (rDorsal-RHD) expressed in *E. coli* cells. As shown in **Figure 9**, rLvDorsal-RHD, but not the control rTrx, effectively retarded the mobility of the bio-labeled probes 1 and 2. The DNA/protein complex was markedly reduced by the competitive unlabeled probe 1 at the level of 10×, 50× and 100×. Contrarily, the DNA/protein complex was only faintly reduced by the competitive 10× and 50× unlabeled probe 2 but markedly reduced by the competitive 100× unlabeled probe 2. These results indicate the specificity of the interaction between rLvDorsal-RHD and the two probes.

### Interaction of LvGSK3β With LvDorsal and LvCactus

**Figure 10:**
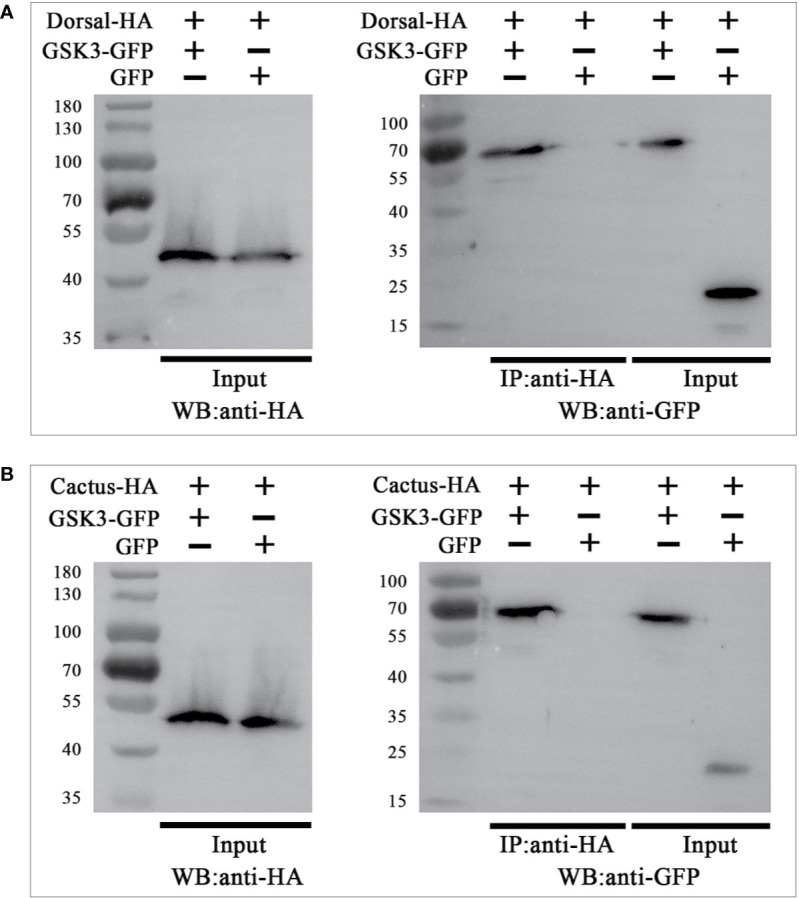
LvGSK3β interacted with LvDorsal **(A)** and LvCactus **(B)**. Co-immunoprecipitation (Co-IP) assays showed that the GFP-tagged LvGSK3β but not the control GFP protein can be co-precipitated by HA-tagged LvDorsal or LvCactus. Input: western-blotting analysis of the input cell lysates (5%) before immunoprecipitation.

To explore whether the interaction of LvGSK3β with LvDorsal or LvCactus occurs in *L. vannamei*, Co-IP analyses were carried out by using the S2 cell line. GFP-tagged LvGSK3β or GFP (as a control) were co-expressed with HA-tagged LvDorsal or HA-tagged LvCactus, respectively. The results showed that both LvDorsal-HA and LvCactus-HA co-precipitated with GFP-tagged LvGSK3β but not the control GFP (**Figures 10A, B**), indicating an interaction of LvGSK3β with LvDorsal and LvCactus.

## Discussion

Previous studies on GSK3, concentrating on vertebrates, have shown that it is a multifunctional enzyme involved in glycogen metabolism, insulin signaling, inflammatory response and innate immunity (38). However, the function of GSK3 in invertebrates has rarely been reported. In the present study, a GSK3β homolog was cloned from *L. vannamei* (LvGSK3β) and was found to be strongly downregulated *in vivo* after WSSV infection, while the inhibition of LvGSK3β significantly increased the survival rate of *L. vannamei* in response to WSSV infection. The effects of inhibition and overexpression of LvGSK3β on NF-κB related genes were the opposite. In addition, interaction between LvDorsal and the LvGSK3β promoter, and the interaction of LvGSK3β with LvDorsal/LvCactus were revealed. All of the results indicated that LvGSK3β has a negative effect on the shrimp by negatively regulating NF-κB activity in *L. vannamei* when it is infected by WSSV.

In vertebrates, GSK3 comprises two homologues, GSK3α and GSK3β, encoded by two similar genes, which share similar Serine/Threonine kinase domains but differ substantially in their termini (39). Unique to the GSK3α is an N-terminal domain consisting of 63 residues that is glycine-rich. Resembling the previously identified GSK3β, *L. vannamei* GSK3β has Ser9 and Tyr216 phosphorylation sites, a STKc_GSK3 domain but no glycine-rich extension at the N-terminus, and the NJ phylogenetic tree showed that LvGSK3β belonged to the cluster of GSK3β homologues from other species, which suggested that LvGSK3β was a member of the β isoform of the GSK3 family in *L. vannamei*. Based on the information on NCBI, most of the GSK3 genes identified were GSK3β in invertebrates including *P. monodon*, *D. pulex* and *E. sinensis*, and GSK3α was only reported in *Drosophila* species. In fact, only GSK3β could be found in *L. vannamei* through scanning the transcriptome and genomic data published on NCBI. The reason why GSK3β but not GSK3α is common in invertebrates has not yet been revealed.

In *E. sinensis*, GSK3β regulated hemocyte phagocytosis in the immune response (40). A previous study reported that LvGSK3β played an important role in shrimp immunomodulation, and shrimp might promote the apoptosis to restrain WSSV infection by inhibition of LvGSK3β (18). Similarly, it was also found that LvGSK3β expression decreased with WSSV challenge, and LvGSK3β inhibition by injection with a specific inhibitor or RNAi reduced the WSSV loads in *L. vannamei* in this study. More intuitively, our study showed that the survival rates of LvGSK3β inhibited *L. vannamei* after the WSSV challenge was greater. All of the results further verified the close relationship between LvGSK3β and WSSV infection, implying the positive function of LvGSK3β on WSSV replication. To our knowledge, this study was a rare report about the role GSK3β plays during viral infection in invertebrates. Comparatively speaking, many more studies about the effect of GSK3β on viral replication have been performed on vertebrates. In humans, there was a significant drop in GSK3β expression in response to Japanese encephalitis virus (JEV) infection (41), and the replication of HCV (15), HIV-1 (16), varicella zoster virus (VZV) (42) and severe acute respiratory syndrome coronavirus (SARS-CoV) (43) was decreased by silencing GSK3β. In addition, GSK3β functioned in inhibiting porcine epidemic diarrhoea virus (PEDV) replication *in vitro* (44). Thus, it can be seen that the effect of GSK3β on viral replication is consistent in vertebrates and invertebrates, indicating the functional conservatism of GSK3β in different animal species.

As an intersection of multiple cell signaling pathways such as Wnt/β-catenin, NF-κB and PI3K/AKT, the function of GSK3β in proliferation, differentiation, apoptosis, and immune responses could not be ignored (10–12). This raises difficulties for clarifying the role of GSK3β and the associated regulatory mechanisms regarding virology. Nevertheless, great progress has been made in humans. For example, the downregulation of GSK3β resulted in a long viral persistence of JEV by enhancing the stability of cyclin D1 (39). For the herpes simplex virus type 1 (HSV-1) -induced Kaposi's sarcoma-associated herpesvirus (KSHV) reactivation, the activation of the MAPK and the PTEN/PI3K/AKT/GSK3β pathways was required (45). VZV benefited its own survival and replication through activating AKT signaling that induces the phosphorylation of GSK3β, which in turn blocks Bad's pro-apoptotic pathway (40). In addition, the decrease of GSK3β expression can also inhibit replication of the influenza virus by positively regulating the virus-induced host cell apoptosis (46). Similar results were presented in the functional study of GSK3β in shrimp. LvGSK3β play an important role in WSSV clearance by mediating apoptosis (18). Consistently, this study found that the expression of LvGSK3β was inhibited in *L. vannamei* upon WSSV challenge and the mortality rates of LvGSK3β-inhibited *L. vannamei* in response to WSSV infection were significantly reduced when compares with the control group. Besides, LvGSK3β expression has a great impact on the expression and transcriptional regulation of LvDorsal, LvCactus and AMP genes, and LvGSK3β interacted with LvDorsal and LvCactus. Furthermore, knockdown of LvDorsal significantly reduced the expression of LvGSK3β. All these results implied that LvGSK3β plays a role during virus infection by regulating NF-κB activity in *L. vannamei* and there was a feedback regulatory loop existed. Briefly speaking, the silence of LvGSK3β by dsRNA resulted in upregulation of LvDorsal and downregulation of LvCactus. The downregulation of LvCactus could indirectly increase the expression of LvDorsal, followed by the increased expression of AMP genes which acted as important effectors of host defense against WSSV (24). At the same time, the increase of LvDorsal brought about the downregulation of LvGSK3β could enhance the effect. When suffer WSSV infection, the inhibition of LvGSK3β expression was also strengthened. All the series of reactions led to the final result was that the survival rates of LvGSK3β-inhibited *L. vannamei* significantly increased when suffer WSSV infection. To our knowledge, although the role of the GSK3β or NF-κB related signaling pathways in viral infection and the control of NF-κB signaling activity by GSKβ has been confirmed separately (18–23, 37), no previous studies have explored the functional mechanisms of GSKβ in viral infection from the perspective of the regulation of NF-κB; therefore, this study is the first such report.

In conclusion, this study revealed that LvGSK3β has a negative effect on *L. vannamei* by negatively regulating NF-κB activity when it is infected by WSSV. The results detail the immune mechanisms and provide a theoretical basis for enhancing the immunity and improving the disease resistance of shrimp, which lays the foundation for the establishment of effective prevention and control of WSS.

## Data Availability Statement

The raw data supporting the conclusions of this article will be made available by the authors, without undue reservation.

## Author Contributions

The experiments were conceived and designed by SZ and LS. SZ, L-LZ, CH, HY, and SY collected samples and performed the experiments. MD, SZ, and LS analyzed the data and completed the writing of the paper. All authors contributed to the article and approved the submitted version.

## Funding

This work was supported by the National Key R&D Program of China (Grant No. 2019YFD0900200), National Natural Science Foundation of China (Grant Nos. 31702377 and 32072988), General Program of Natural Science Foundation of Guangdong Province, China (Grant Nos. 2018A030313963 and 2020A1515010319) and Research Fund Program of Guangdong Provincial Key Laboratory of Marine Resources and Coastal Engineering.

## Conflict of Interest

The authors declare that the research was conducted in the absence of any commercial or financial relationships that could be construed as a potential conflict of interest.
